# Prx1 Regulates Thapsigargin-Mediated UPR Activation and Apoptosis

**DOI:** 10.3390/genes13112033

**Published:** 2022-11-04

**Authors:** Eun-Kyung Kim, Yosup Kim, Jun Young Yang, Ho Hee Jang

**Affiliations:** Department of Biochemistry, College of Medicine, Lee Gil Ya Cancer and Diabetes Institute, Gachon University, Incheon 21999, Korea

**Keywords:** peroxiredoxin, endoplasmic reticulum stress, reactive oxygen species, unfolded protein response signaling, apoptosis

## Abstract

Endoplasmic reticulum (ER) stress activates the unfolded protein response (UPR) signaling via the accumulation of unfolded and misfolded proteins. ER stress leads to the production of reactive oxygen species (ROS), which are necessary to maintain redox homeostasis in the ER. Although peroxiredoxin 1 (Prx1) is an antioxidant enzyme that regulates intracellular ROS levels, the link between Prx1 and ER stress remains unclear. In this study, we investigated the role of Prx1 in X-box binding protein 1 (XBP-1) activation, the C/EBP homologous protein (CHOP) pathway, and apoptosis in response to ER stress. We observed that Prx1 overexpression inhibited the nuclear localization of XBP-1 and the expression of XBP-1 target genes and CHOP after thapsigargin (Tg) treatment to induce ER stress. In addition, Prx1 inhibited apoptosis and ROS production during ER stress. The ROS scavenger inhibited ER stress-induced apoptosis but did not affect XBP-1 activation and CHOP expression. Therefore, the biological role of Prx1 in ER stress may have important implications for ER stress-related diseases.

## 1. Introduction

Peroxiredoxins (Prxs) are a ubiquitous family of antioxidant enzymes that reduce peroxides [1]. Prx1 is a member of the 2-Cys Prx family and is an abundant protein that is present mainly in cytoplasm. Under stress conditions, Prx1 undergoes a structural transition to function as a chaperone from a peroxidase [2]. In addition, Prx1 shows abnormal expression in several human cancers, including lung, breast, and prostate cancers, as well as modulates various ROS-mediated signaling pathways, such as phosphatase and tensin homolog (PTEN) and mammalian Ste20-like kinase-1 (MST) [1,3,4]. Prx1 acts as a tumor suppressor that inhibits tumorigenesis and promotes tumor cell death by interacting with c-Myc and as an oncogene that inhibits tumor cell death by interacting with nuclear factor kappa B (NF-κB) and the androgen receptor (AR) [5,6,7].

The ER is an organelle involved in the folding and maturation of newly synthesized proteins to be secreted [8]. When unfolded proteins accumulate in the ER or when calcium is deficient, ER stress is induced, and cells activate an adaptive mechanism known as the UPR to overcome it [9]. The UPR activation depends on three ER stress sensors: inositol-requiring enzyme 1α (IRE1α), activating transcription factor 6 (ATF6), and protein kinase RNA-like ER kinase (PERK) [10,11]. Under ER stress, IRE1α oligomerizes and autophosphorylates to activate its RNase domain [10]. Active IRE1α removes the intron of XBP-1 via an unconventional splicing reaction, resulting in the production of the functional transcription factor XBP-1s [10]. XBP-1s induces the expression of ER chaperones and ER-associated degradation (ERAD) genes to restore ER homeostasis [10]. Under ER stress, ATF6 is trafficked from the ER to the Golgi and cleaved by site-1 and site-2 proteases (S1P and S2P, respectively), releasing the transcription domain from the membrane [11]. Cleaved ATF6 translocates to the nucleus and activates the expression of UPR target genes [11]. Finally, dimerization and autophosphorylation of PERK occur to favor the phosphorylation of eukaryotic translation initiation factor 2α (eIF2α) in response to ER stress [12,13]. Phosphorylated eIF2α inhibits protein synthesis, resulting in the reduction of protein influx into ER [13]. Furthermore, it upregulates ATF4 to restore ER homeostasis or initiates the apoptosis pathway via the expression of UPR target genes [14,15].

Moreover, under prolonged and irreversible ER stress, the UPR mechanism cannot restore ER homeostasis, and the cells switch to proapoptotic signaling to induce apoptosis in damaged cells [11]. Response to ER stress necessitates the coordination of proapoptotic and prosurvival mechanisms, and the key factor responsible for this is the induction of the transcription factor CCAAT/enhancer-binding protein (C/EBP)-homologous protein (CHOP), regulated by ATF4 [16,17]. In response to ER stress, CHOP inhibits the expression of BCL-2 family members and induces apoptosis in cells [18]. The apoptotic pathway is particularly important for ROS-mediated apoptosis [19]. Thus, ER stress results in the accumulation of reactive oxygen species (ROS), leading to apoptosis and malfunctioning [19].

The PDI–ERO1 cycle generates ROS accumulation, whereas ER-localized Prx4 reduces ER stress-induced ROS production [20,21]. In addition, ER stress-induced UPR signaling was activated in Prx1 knockout mice [22]. Although Prx1 is involved in ER stress-induced UPR signaling, the association between Prx1 and ROS remains unclear. The present study investigated the role of Prx1 in XBP-1 activation, the CHOP pathway, and apoptosis, as well as its link with ROS in response to ER stress.

## 2. Materials and Methods

*Materials.* The human full-length Prx1 gene sequence was amplified by PCR and ligated into a pCS4-3Myc-plasmid. Primary antibodies against c-Myc, XBP-1s, PARP1, and BAX were obtained from Santa Cruz Biotechnology (Dallas, TX, USA). The anti-β-actin antibody was purchased from Millipore (Billerica, MA, USA). The anti-Prx1 antibody was manufactured by AB Frontier (Seoul, South Korea). The ER stress inducer Tg was obtained from Sigma Aldrich (St. Louis, MO, USA).

*Cell culture and transfection.* HEK293 and HeLa cells were cultured in Dulbecco’s Modified Eagle’s Medium (DMEM) (WelGENE, Daegu, South of Korea) containing 10% fetal bovine serum (FBS) (Capricorn, Germany) and 1% penicillin/streptomycin (WelGENE, Daegu, South of Korea). Cells were incubated at 37 °C in 5% CO_2_ humidified air. Cells were seeded at a density of 1.25 × 10^5^ cells on a 6 cm dish. After incubation for 16 h, the cells were transfected using the TransIT-X2^®^ Transfection Kit (Mirus, MIR6000, Madison, WI, USA) as per the manufacturer’s instructions. After incubation for 48 h, cells transfected with plasmid or siRNA were harvested. Prx1 was overexpressed using the plasmid pCS4-3Myc-Prx1. Prx1 levels were knocked down in HEK293 cells using siRNA. Both siPrx1 (sc-36177) and against a scrambled sequence (siCON) (sc-37007) were purchased from Santa Cruz Biotechnology, Inc. (Santa Cruz, CA, USA). Tg was treated after 16 h of serum starvation.

*RNA isolation and XBP-1 splicing assay*. HEK293 cells, transfected with or without ER stress inducer, were harvested. Total RNA was isolated from the harvested cells using TRIzol reagent (Invitrogen, Carlsbad, CA, USA), as per the manufacturer’s instructions, and was quantified. Total RNA was treated with DNase I (Thermo Fisher Scientific, Waltham, MA, USA) at 37 °C for 30 min and inactivated at 72 °C for 10 min. Purified total RNA (1 µg) was reverse-transcribed and amplified by PCR using 2X TOPsimple^TM^ Dye MIX-Tenuto (Enzynomics, Daejeon, South Korea) following the manufacturer’s protocol for reverse transcription. The XBP-1 gene was amplified after 30 cycles. The following RT–PCR primer pairs were used: human *XBP-1* (gene ID: 7494), 5′-TTACGAGAGAAAACTCATGGCC-3′ and 5′-GGGTCCAAGTTGTCCAGAATGC-3′; human *ACTB* (gene ID: 60), 5′-TCCCTGGAGAAGAGCTACGA-3′ and 5′-AGCACTGTGTTGGCGTACAG-3′. The target mRNA was detected and captured using a Gel Doc XR system (Bio-Rad, Hercules, CA, USA).

*Nuclear/cytosol fractioning*. HEK293 cells were transfected with pCS4-3Myc-Prx1 or exposed to 5 mM N-acetylcysteine (NAC) and/or 300 nM Tg for 12 h. Harvested HEK293 pellets were incubated in a digitonin-containing buffer (150 mM NaCl, 50 mM HEPES, pH 7.4, 100 μg/mL digitonin, and 5 mM EDTA) with a protease inhibitor cocktail (GenDEPOT, Katy, TX, USA) for 10 min at 4 °C and centrifuged for 10 min at 2000× *g*. Cytosolic supernatants were cleared by centrifugation at 20,000× *g* for 5 min. The nuclear pellet was washed with PBS and lysed with RIPA buffer (20 mM HEPES (pH 7.5), 150 mM NaCl, 5 mM EDTA, 10% (*v*/*v*) glycerol, and 1% (*v*/*v*) Triton X-100) containing a protease inhibitor cocktail (GenDEPOT, TX, USA). Finally, the nuclear and cytoplasmic fractions were subjected to Western blot analysis.

*Western blot.* Harvested HEK293 cells were lysed with RIPA buffer containing a protease inhibitor cocktail (GenDEPOT, Katy, TX, USA) with vortexing. About 30 μg of lysate was mixed with reducing loading buffer, boiled at 100 °C for 5 min, subjected to SDS–PAGE method, and electro-transferred onto nitrocellulose membranes (NC membrane) (GE Healthcare, Milwaukee, WI, USA). Transferred membranes were immersed in TBST solution (Tris-buffered saline with 0.1% (*v*/*v*) Tween-20) containing 8% (*w*/*v*) nonfat milk for 30 min at room temperature to block nonspecific binding sites in blots. Blots were incubated with primary antibodies (XBP-1 at 1:1000, histone H3 at 1:1000, PARP-1 at 1:1000, Bax at 1:1000, Myc at 1:1000, Prx1 at 1:1000, and β-actin at 1:1000), kept overnight at 4 °C, washed with TBST at room temperature, incubated with horseradish peroxidase (HRP)-conjugated secondary antibodies (1:3000) for 2 h at room temperature, washed thrice with TBST, and detected using ECL reagents (Amersham, Piscataway, NJ, USA).

*Quantitative reverse transcription PCR*. The synthesized cDNA was quantified using real-time PCR with SYBR Premix Ex TaqII (Takara, Japan) and determined using an iCycler (Bio-Rad, Hercules, CA, USA). The following primer sets were used: human *ERDJ4* (Gene ID: 4189), 5′-AAGTTGGCCATGAAGTACCA-3′ and 5′-TGGCGTGTCTGGAAATGATT-3′; human *HERP* (Gene ID: 9709), 5′-CTCAAGTGGTTGTTAATCCT-3′ and 5′-GAGGATACTGAGAAAAACAG-3′; human *CHOP* (Gene ID: 1649), 5′-GATTCTTCCTCTTCATTTCC-3′ and 5′-CAGGAGGTGAAACATAGGTA-3′; human Prx1 (Gene ID: 5052), 5′-GACCCATGAACATTCCTTTGGT-3′ and 5′-CAGCCTTTAAGACCCCATAATCC-3′; and human *GAPDH* (Gene ID: 2597), 5′-AGGGCTGCTTTTAACTCTGGT-3′ and 5′-CCCCACTTGATTTTGGAGGGA-3′. The human GAPDH gene was used as a control to normalize RNA expression. qPCR was performed thrice for each sample. Gene expression was calculated using the ΔΔ^Ct^ method.

*Annexin V assay*. HEK293 cells were induced with overexpressed Prx1 or treated with 300 nM Tg for 12 h. HEK293 cells were treated with trypsin and harvested. The harvested cells were washed thrice with PBS and centrifuged at 3000 rpm for 5 min. annexin V analysis was performed using the EzWay annexin V-FITC Apoptosis Detection Kit (Koma Biotech, Seoul, South Korea), as per the manufacturer’s protocol. Flow cytometry was performed using BD FACSCalibur (BD Biosciences, Durham, NC, USA).

*Immunofluorescence.* HeLa cells were incubated at a density of 2 × 10^4^ cells per 24 well on glass coverslips and transfected with overexpressed Prx1 using pCS4-3Myc-Prx1. Overexpressed Prx1 in HeLa cells were treated with 300 nM Tg for 12 h. Cells were fixed with 4% (*v*/*v*) formaldehyde in PBS for 15 min. Fixated cells were washed thrice with 0.1 M glycine in PBS to quench autofluorescence. Cells were permeabilized with 0.2% (*v*/*v*) Triton X-100 for 10 min. After washing with PBS, cells were incubated with 5% (*w*/*v*) bovine serum albumin (BSA; Bovogen Biologicals, Victoria, Australia) in PBS for 30 min. The slides were then incubated with an anti-Prx1 (1:100) antibody at 4 °C for 16 h. After washing thrice with PBS, anti-mouse IgG Alexa Fluor 488 antibody (1:500, A11001, Invitrogen, CA, USA) was added and the nuclei were stained using Vectashield mounting medium for fluorescence with DAPI (H- 1200, Vector Laboratories, Burlingame, CA, USA). Images were obtained using an LSM confocal microscope (Zeiss, Germany).

*DCF-DA staining*. HeLa cells were induced with overexpressed Prx1 or treated with 5 mM NAC and/or 300 nM Tg for 12 h. These cells were incubated with 20 μM of 2,7-dichlorofluorescein diacetate (DCF-DA) (Invitrogen, CA, USA) in the dark at 37 °C for 30 min to measure intracellular ROS levels. The cells were washed with PBS once. Next, intracellular ROS levels were evaluated by calculating the relative fluorescence intensity (RFI) from each group at 488 nm using a confocal laser microscope.

*Statistical analysis*. All experiments were performed thrice, and data are presented as the mean ± standard deviation (SD). Comparisons between two groups were analyzed using Student’s *t*-test. Differences were considered statistically significant at * *p* < 0.05, ** *p* < 0.01, and *** *p* < 0.001.

## 3. Results

### 3.1. ER Stress Induces UPR Activation, CHOP Pathway, and Apoptosis in HEK293 Cells

**Figure 1:**
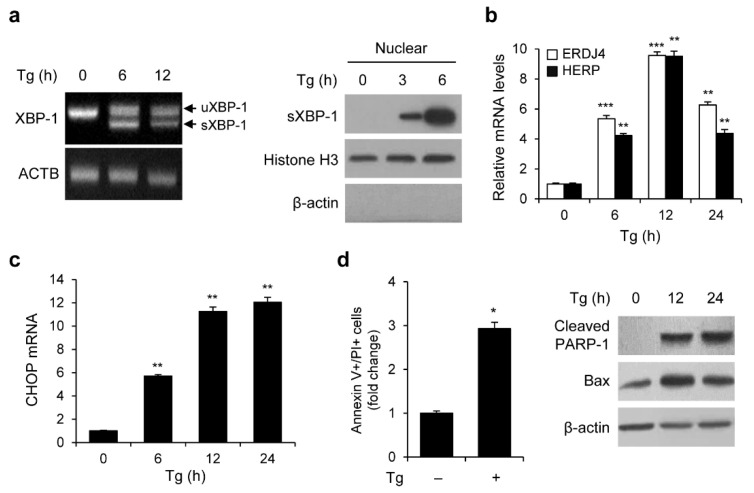
ER stress induces UPR activation, CHOP pathway, and apoptosis. (**a**) RT-PCR of s*XBP-1* and u*XBP-1* in HEK293 cells treated with 300 nM Tg at indicated times. Proteins in nuclear fractions were analyzed by Western blotting (WB). (**b**,**c**) The mRNA expression levels of *ERDJ4* and *HERP* (**b**) or *CHOP* (**c**) were analyzed using qRT-PCR. (**d**) Apoptosis assay was performed after HEK293 cells were treated with 300 nM Tg for 24 h. Quantification of annexin V/PI positive cells was expressed as fold change compared to control cells. The protein levels of cleaved PARP-1 and Bax were analyzed using WB. (* *p* < 0.05, ** *p* < 0.01 and *** *p* < 0.001).

The ER calcium pump is inhibited by the ER stress inducer Tg, which leads to ER malfunction [23]. We investigated XBP-1 splicing and nuclear translocation in HEK293 cells to test whether Tg induces UPR activation. The cells were treated with Tg in a time-dependent manner, and RT-PCR was used to quantify the mRNA levels of unspliced *XBP-1* (uXBP-1) and spliced *XBP-1* (sXBP-1) (Figure 1a). The sXBP-1 mRNA levels were upregulated in response to Tg treatment (Figure 1a). Additionally, we observed that the amount of XBP-1 in the nuclear fraction increased after Tg treatment in a time-dependent manner (Figure 1b). sXBP-1 regulates several UPR target genes, including ER chaperones (BIP/GRP78, ERDJ4, ERDJ5, HEDJ, GRP58, and PDIP5) and ERAD components (EDEM, HERP, and p58IPK) [24]. The mRNA expression levels of *ERDJ4* and *HERP* in response to Tg were measured using qRT-PCR. The mRNA expression levels of *ERDJ4* and *HERP* increased by up to nine times after 12 h of Tg treatment (Figure 1b).

ER stress not only induces apoptosis through activation of the CHOP pathway but also directly induces apoptosis. Therefore, we examined whether Tg treatment activates such a pathway in HEK293 cells. The cells were treated with Tg for 6, 12, or 24 h, and *CHOP* mRNA levels were determined using qRT-PCR. *CHOP* mRNA levels were upregulated in response to Tg treatment (Figure 1c). Apoptotic cells treated with Tg were measured by flow cytometry. After 24 h of Tg treatment, annexin V-PI-positive cells were increased approximately 3-fold compared to untreated cells (Figure 1d). In addition, Western blotting demonstrated that Tg raised the levels of PARP-1 cleavage and BAX in a time-dependent manner (Figure 1d). These results indicate that the ER stress inducer, Tg, efficiently triggers UPR activation, the CHOP pathway, and apoptosis in HEK293 cells.

### 3.2. Prx1 Regulates ER Stress-Induced UPR Activation, CHOP Pathway, and Apoptosis

**Figure 2:**
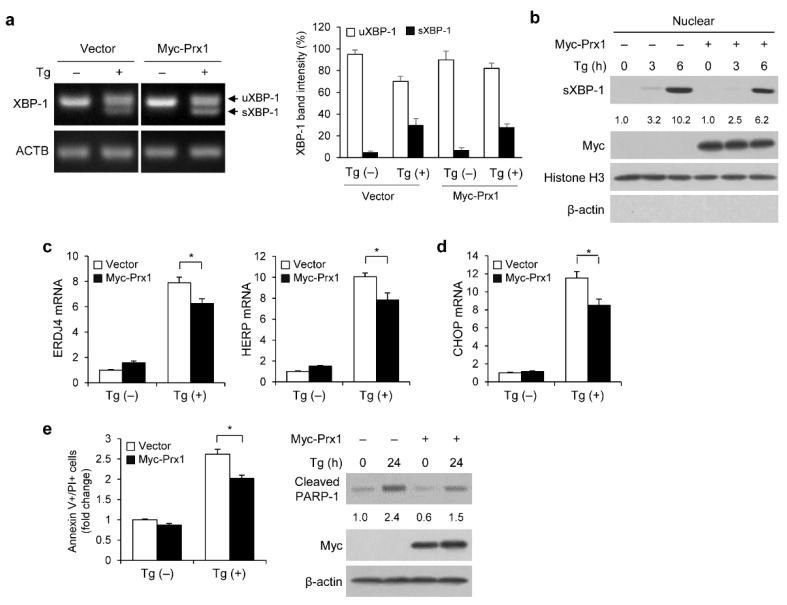
Prx1 inhibits ER stress-induced UPR activation, CHOP pathway, and apoptosis. HEK293 cells were transfected with vector or Myc-Prx1. (**a**) RT-PCR of spliced XBP-1 in transfected HEK293 cells treated with or without 300 nM Tg for 24 h. The band intensity was measured by ImageJ. (**b**) WB of nuclear fraction in transfected HEK293 cells treated with 300 nM Tg for the indicated times. The intensity of sXBP-1 bands was normalized to Histone H3 levls and was quantified using ImageJ software. (**c**,**d**) Transfected HEK293 cells were incubated with or without 300 nM Tg for 12 h. The mRNA expression levels of *ERDJ4* and *HERP* (**c**) or *CHOP* (**d**) were analyzed using qRT-PCR. The mRNA levels are shown as fold changes as compared to control vector cells. (**e**) Apoptosis assay of overexpressed Prx1 in HEK293 cells incubated with or without 300 nM Tg for 24 h using the annexin V/PI staining assay. Quantification of annexin V/PI positive cells was expressed as fold change compared to control vector cells. The cleaved PARP-1 protein levels were analyzed using WB. The band intensity of the cleaved PARP-1 level was normalized to β-actin using ImageJ software. Data are indicated as the mean ± SD of three independent experiments (* *p* < 0.05).

To determine the regulatory effect of Prx1 on ER stress-induced UPR signaling, we examined the effect of exogenous Prx1 overexpression in HEK293 cells. After transfecting cells with a control vector or Myc-Prx1, we measured the variation in XBP-1 splicing, the nuclear translocation of sXBP-1, and the expression levels of sXBP-1 target genes. In XBP-1 splicing, the mRNA levels of sXBP-1 in response to Tg treatment were similar to those of Prx1 overexpression and the vector (Figure 2a). Prx1 overexpression, however, inhibited nuclear translocation of sXBP-1 after receiving Tg treatment (Figure 2b). Similar to this finding, Prx1 overexpression inhibited the Tg-induced upregulation of *ERDJ4* and *HERP* mRNA levels (Figure 2c). Furthermore, we investigated whether Prx1 regulates the CHOP pathway and apoptosis during ER stress. After transfecting the cells with a control vector or Myc-Prx1, we treated them with Tg. As shown in Figure 2d, 12 h after Tg treatment, CHOP mRNA levels increased approximately 11 times. However, compared to control cells, Prx1-overexpressed cells had 26% lower CHOP mRNA levels.

To examine the involvement of Prx1 in Tg-induced apoptosis, we measured the number of apoptotic cells using flow cytometry. When HEK293 cells were treated with Tg, the proportion of apoptotic cells increased by 2.6-fold. After 24 h of Tg treatment, the proportion of apoptotic cells was 23% lower in Prx1-overexpressed cells than that in control cells (2.02 ± 0.08 vs. 2.62 ± 0.12, *p* < 0.05) (Figure 2e). Similarly, Prx1 overexpression inhibited Tg-induced PARP-1 cleavage (Figure 2e). These results indicated that Prx1 overexpression inhibits UPR activation, CHOP expression, and apoptosis. 

**Figure 3:**
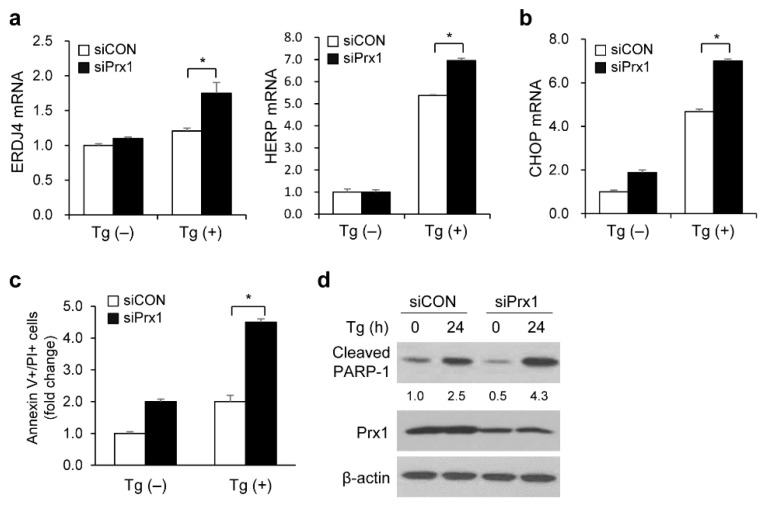
Prx1 knockdown accelerates ER stress-induced UPR activation, CHOP pathway, and apoptosis. (**a**) Prx1 knockdown accelerates the expression of *ERDJ4* and *HERP* mRNA with Tg treatment. HEK293 cells transfected with siRNA against a siCON or siPrx1 were incubated with or without 300 nM Tg for 12 h. The mRNA levels of *ERDJ4* and *HERP* were analyzed using qRT-PCR. The mRNA levels are shown as fold changes compared with control siCON groups. (**b**) Prx1 knockdown enhanced the expression of Tg-induced *CHOP* mRNA. HEK293 cells transfected with siRNA against a siCON or siPrx1 were incubated with or without 300 nM Tg for 12 h. The mRNA level of *CHOP* was analyzed using qRT-PCR. The mRNA levels are shown as fold changes compared with untreated siCON groups. (**c**,**d**) Prx1 knockdown induced apoptosis with Tg treatment. HEK293 cells transfected with siRNA against siCON or siPrx1 were incubated with or without 300 nM Tg for 12 h. (**c**) The percentage of apoptotic cells was determined using the annexin V/PI staining assay. Quantification of annexin V/PI positive cells was expressed as fold change compared to control siCON groups. (**d**) The protein levels of cleaved PARP-1 and Prx1 were analyzed using Western blotting. The band intensity of the cleaved PARP-1 level was normalized to β-actin using ImageJ software. Data are indicated as the mean ± SD of three independent experiments (* *p* < 0.05).

To confirm whether Prx1 mediated regulation of Tg-induced apoptosis via UPR signaling, we induced Prx1 knockdown using siRNA and analyzed apoptosis and mRNA levels of *ERDJ4* and *HERP*. Prx1 knockdown increased Tg-induced upregulation of *ERDJ4* and *HERP* mRNA levels (Figure 3a). In addition, CHOP mRNA expression levels were upregulated by Prx1 silencing after Tg treatment (Figure 3b). The annexin V/PI staining assay demonstrated that Tg-mediated apoptotic cell induction was significantly higher in Prx1-depleted cells (Figure 3c). Furthermore, we showed that depletion of Prx1 induced the cleavage of PARP-1 protein compared to Tg-treated control cells (Figure 3d). Consequently, Prx1 is a crucial regulator of ER stress-induced UPR signaling.

### 3.3. Prx1 Overexpression Inhibits ER Stress-Induced ROS Production

**Figure 4:**
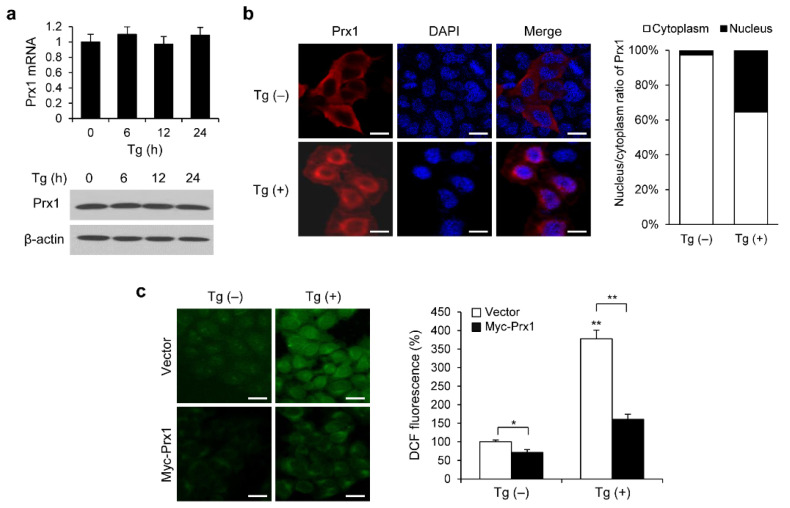
Prx1 overexpression inhibits ER stress-induced ROS production. (**a**) qRT-PCR and WB were performed to detect *PRDX1* mRNA and protein expression levels in HEK293 cells treated with 300 nM Tg. (**b**) Immunofluorescence of HeLa cells treated with 300 nM Tg for 12 h. Scale bar, 20 μm. The ratio (%) of the average intensities of the Prx1 signals in the nucleus and cytoplasm was determined using ImageJ software. (**c**) DCF-DA staining of HeLa cells with control vector or Myc-Prx1 exposed to 300 nM Tg for 12 h. The DCF fluorescence intensity was assessed using ImageJ software. Scale bar, 20 μm. Data are expressed as the mean ± SD of three independent experiments (* *p* < 0.05 and ** *p* < 0.01).

We investigated the effect of Tg on the expression levels and localization of Prx1 in HEK293 cells to determine if the changes in Prx1 expression and localization were associated with the ER stress-induced UPR signaling. After the cells were treated with Tg for 0, 6, 12, or 24 h, the Prx1 mRNA and protein levels were measured using qPCR and Western blotting, respectively. As shown in Figure 4a, the expression levels of Prx1 mRNA and protein did not alter in response to Tg treatment. To study the Prx1 localization, HeLa cells were exposed to Tg for 12 h and assessed via immunofluorescence. Prx1 was mainly distributed in the cytoplasm of the control cells; however, it was increased in the nucleus of Tg-treated cells (Figure 4b). Thus, the Tg-induced effect of Prx1 may be involved in the control of ER stress-induced UPR signaling. 

To investigate the effect of Prx1 on ER stress-induced ROS accumulation, we examined Tg-induced ROS production in Prx1 overexpression cells using DCF staining. After the overexpression of Prx1 in HeLa cells, Tg was treated for 12 h. In the absence of Tg treatment, overexpression of Prx1 reduced intracellular ROS levels. In comparison to control cells, DCF fluorescence was 3.77-fold higher in Tg-treated cells (377 ± 22 vs. 100 ± 5, *p* < 0.01). In Prx1-overexpressed cells, DCF fluorescence in Tg-treated cells was 2.25-fold higher than that in untreated cells (159 ± 12 vs. 71 ± 5, *p* < 0.01). Moreover, ER stress-induced ROS production was 40% lower in Prx1-overexpressed cells than in control cells (Figure 4c). These results indicate that Prx1 is involved in the suppression of ROS production during ER stress. 

### 3.4. ER Stress-Induced ROS Production Is Associated with Apoptosis but Does Not Affect UPR Signaling

**Figure 5:**
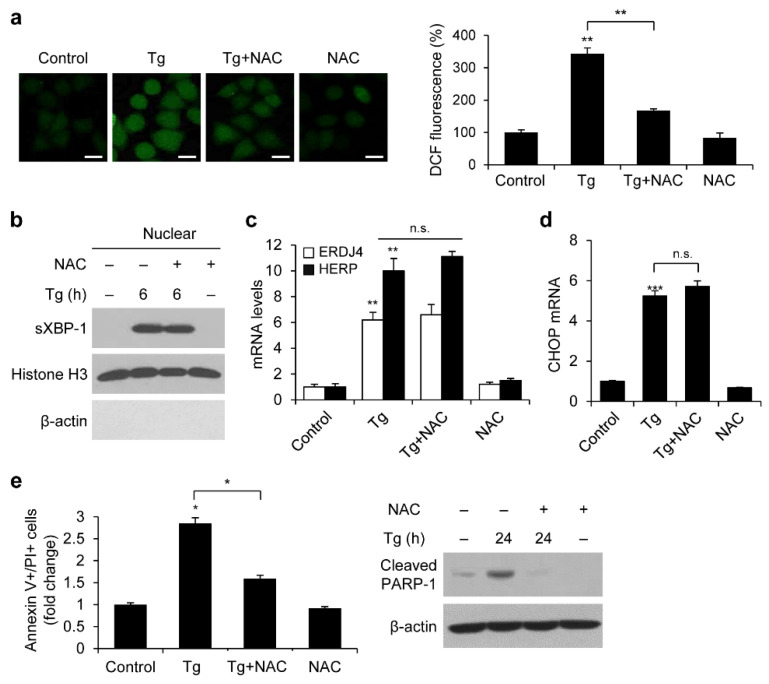
ROS production by ER stress is associated with enhanced apoptosis; however, it does not affect UPR and CHOP activation. (**a**) HeLa cells were treated with 5 mM NAC or/and 300 nM Tg for 12 h. Intracellular ROS were measured by DCF-DA staining using confocal microscopy. The DCF fluorescence intensity was assessed using Image J software. Scale bar, 20 μm. (**b**) WB of nuclear fractions in HEK293 cells treated with 5 mM NAC or/and 300 nM Tg for the indicated time. (**c**,**d**) The mRNA levels of *ERDJ4* and *HERP* (**c**) or *CHOP* (**d**) in HEK293 cells treated with 5 mM NAC or/and 300 nM Tg for 12 h analyzed using qRT-PCR. The mRNA levels are shown as fold changes compared with control vector cells. (**e**) WB of cleaved PARP-1 in HEK293 cells treated with 5 mM NAC or/and 300 nM Tg for 24 h. Data are expressed as the mean ± SD of three independent experiments (* *p* < 0.05, ** *p* < 0.01, and *** *p* < 0.001). n.s. not significant.

Prx1 not only inhibited ER stress-induced ROS production but also XBP1 and CHOP pathways, as well as apoptosis (Figure 2 and Figure 3). Therefore, to determine whether the inhibition of ROS production by Prx1 affects UPR signaling and apoptosis during ER stress, we examined the effect of the ROS scavenger NAC on HEK293 cells. First, we analyzed the DCF fluorescence intensity following NAC pretreatment to determine intracellular ROS production by ER stress. The cells treated with Tg for 12 h increased intracellular ROS levels by 3.4-fold, whereas NAC-treated cells inhibited ROS production (Figure 5a). Second, we examined whether the NAC-mediated reduction in ROS could restore XBP-1 and CHOP expression. However, NAC was unable to inhibit the Tg-induced nuclear translocation of sXBP1 (Figure 5b). NAC treatment also failed to suppress the Tg-induced expression of its target genes (*ERDJ4* and *HERP*) and *CHOP* (Figure 5c,d). These results indicated that ROS is neither involved in ER stress-induced UPR activation nor CHOP expression.

We further demonstrated apoptosis in HEK293 cells by pretreating them with NAC before Tg treatment to investigate whether ROS production is associated with ER stress-induced apoptosis. NAC pretreatment efficiently reduced the proportion of apoptotic cells compared to Tg-treated cells (Figure 5e). In addition, Western blot analysis showed a significant decrease in cleaved PARP-1 after NAC pretreatment. These findings showed that ER stress induces apoptosis through ROS production, which can be prevented by the ROS scavenger NAC.

**Figure 6:**
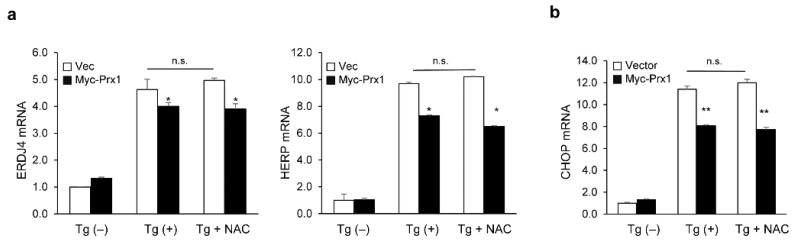
Prx1 regulates UPR activation in a ROS-independent manner. Transfected HEK293 cells were incubated with 5 mM NAC or/and 300 nM for 12 h. The mRNA levels of *ERDJ4* and *HERP* (**a**) or *CHOP* (**b**) were analyzed using qRT-PCR. The mRNA levels are shown as fold changes compared with control vector cells. Data are expressed as the mean ± SD of three independent experiments (* *p* < 0.05 and ** *p* < 0.01). n.s. not significant.

To determine whether Prx1 is ROS-independently involved in regulating UPR activation, we investigated the levels of *ERDJ4*, *HERP*, and *CHOP* with or without NAC treatment under ER stress. NAC treatment had no effect on UPR activation and CHOP expression inhibited by Prx1 (Figure 6). Therefore, these results indicated that Prx1 regulates ROS-independent UPR activation during ER stress.

## 4. Discussion

This study investigated the role of Prx1 and ROS in response to ER stress. We demonstrated that ER stress induced Prx1 to inhibit the activation of UPR signaling, CHOP expression, and apoptosis. Prx1 may control ER stress-induced UPR and CHOP activation by mechanisms other than the regulation of ROS levels. 

Moreover, we discovered that apoptosis enhanced ER stress-induced ROS production; however, this did not contribute to the activation of UPR signaling. During protein folding in the lumen of the ER, ROS production is increased by protein disulfide isomerase (PDI), ER membrane-associated protein, and endoplasmic reticulum oxidoreductin-1 (ERO1) [21]. In some cases, ROS generation triggers UPR activation [25]. According to our findings, Prx1 overexpression suppresses ROS generation and UPR activation. Although apoptosis was reduced by ER stress-induced ROS, the ROS scavenger NAC did not affect the activation of the UPR and CHOP pathways. These results suggest that the peroxidase activity of Prx1 is not involved in the signal activation of UPR and CHOP and directly inhibits apoptosis by reducing ER stress-induced ROS. These findings demonstrate that the regulation of UPR activation by Prx1 is a novel mechanism that is not mediated by ROS. This possibility is supported by the variation in the cellular location of Prx1 through immunofluorescence but not its Tg-treated mRNA and protein expression levels. 

Furthermore, Prx1 is entirely found in the cytosol under normal conditions [2]. When exposed to Tg, Prx1 translocates into the nucleus. This finding supports the hypothesis that Prx1 regulates UPR signaling via other mechanisms. During ER stress, cytosolic Prx1 suppresses the nuclear translocation of XBP via unknown mechanisms, such as physical interaction, and can inhibit UPR signaling. In addition, nuclear-translocated Prx1 can inhibit UPR signaling through modulation of ER stress-related factors, such as CHOP.

Previous studies have revealed that the translocation of XBP-1 was interrupted by CDK5 and PI3K [26,27]. According to our data, Prx1 does not affect the splicing of XBP1 but inhibits migration to the nucleus in the presence of Tg. This result suggested that nuclear Prx1 may directly or indirectly affect XBP1 regulation in response to Tg-induced ER stress.

A previous study showed that when oxidative protein folding occurs in ER lumen, Prx4 eliminated the ER oxidase, ERO1-induced hydrogen peroxide, and protects cells against ER-stress-induced cell death [28,29]. Based on our data, we demonstrated that Prx1, unlike Prx4, inhibited ER stress-associated activation of UPR signaling but also ER stress-induced apoptosis.

## 5. Conclusions

Our findings emphasize that Prx1 responds to ER stress via mechanisms other than the elimination of ER stress-induced ROS. Finally, this study revealed that Prx1 plays a novel role as a therapeutic target for ER stress and is the main regulator of UPR signaling and apoptosis. Further studies should be conducted to investigate the effect of Prx1 on the interaction and expression of UPR signaling and the dependence of Prx1 on subcellular location through various experimental approaches.

## Data Availability

Not applicable.
